# Targeting Catalase but Not Peroxiredoxins Enhances Arsenic Trioxide-Induced Apoptosis in K562 Cells

**DOI:** 10.1371/journal.pone.0104985

**Published:** 2014-08-12

**Authors:** Li-Li Song, Yao-Yao Tu, Li Xia, Wei-Wei Wang, Wei Wei, Chun-Min Ma, Dong-Hua Wen, Hu Lei, Han-Zhang Xu, Ying-Li Wu

**Affiliations:** Department of Pathophysiology, Chemical Biology Division of Shanghai Universities E-Institutes, Key Laboratory of Cell Differentiation and Apoptosis of the Chinese Ministry of Education, Shanghai Jiao Tong University School of Medicine, Shanghai, China; Università degli Studi di Firenze, Italy

## Abstract

Despite considerable efficacy of arsenic trioxide (As_2_O_3_) in acute promyelocytic leukemia (APL) treatment, other non-APL leukemias, such as chronic myeloid leukemia (CML), are less sensitive to As_2_O_3_ treatment. However, the underlying mechanism is not well understood. Here we show that relative As_2_O_3_-resistant K562 cells have significantly lower ROS levels than As_2_O_3_-sensitive NB4 cells. We compared the expression of several antioxidant enzymes in these two cell lines and found that peroxiredoxin 1/2/6 and catalase are expressed at high levels in K562 cells. We further investigated the possible role of peroxirdoxin 1/2/6 and catalase in determining the cellular sensitivity to As_2_O_3_. Interestingly, knockdown of peroxiredoxin 1/2/6 did not increase the susceptibility of K562 cells to As_2_O_3_. On the contrary, knockdown of catalase markedly enhanced As_2_O_3_-induced apoptosis. In addition, we provide evidence that overexpression of BCR/ABL cannot increase the expression of PRDX 1/2/6 and catalase. The current study reveals that the functional role of antioxidant enzymes is cellular context and treatment agents dependent; targeting catalase may represent a novel strategy to improve the efficacy of As_2_O_3_ in CML treatment.

## Introduction

As_2_O_3_ has long been used therapeutically in China and in the Western World [1]. For example, Fowler solution (potassium arsenite), has been used for the treatment of chronic myeloid leukemia (CML), syphilis, ulcer, etc. in the 18^th^ and 19^th^ centuries [2]. However, due to the concerns about toxicity and carcinogenicity, the medical use of As_2_O_3_ was discontinued_._ After the discovery that As_2_O_3_ is an efficient drug for the treatment of acute promyelocytic leukemia (APL), As_2_O_3_ was reintroduced in current therapeutic concepts [3]–[4].

Accumulating reports have demonstrated that As_2_O_3_ can interfere with a variety of cellular processes by targeting numerous different intracellular molecules, thereby disrupting key signal transduction mechanisms and resulting in cell death. For instance, generation of reactive oxygen species (ROS) [5], activation of JNK [6], inhibition of NF-κB [7], inhibition of angiogenesis [8], and down-regulation of telomerase [9], Bcl-2 [10], have been shown to contribute to As_2_O_3_-induced cell death. These findings emphasize the importance of understanding how the difference in cell type or cellular environment might affect the actions of As_2_O_3_.

The anti-APL activity of As_2_O_3_ has been mainly attributed to the degradation of the fusion oncoprotein PML-RARα, which results from the t(15;17) chromosome translocation [11]–[14]. Interestingly, As_2_O_3_ can also induce the degradation of BCR/ABL [15]–[16], the pivotal oncogenic fusion protein in CML, which arises from the t(9;22) chromosome translocation [17]. Targeting inhibition of BCR/ABL kinase activity by Gleevec induces cell death in CML cells and remission in CML patients [18]. Despite of this, APL cells are more sensitive to As_2_O_3_-induced cell death than CML cells, indicating that other factors, beyond these two oncoproteins, may responsible for their sensitivity to As_2_O_3_.

In this study, we found that the As_2_O_3_-resistant K562 cells have a much lower level of ROS than the As_2_O_3_-sensitive NB4 cells. In addition, several antioxidant enzymes, such as catalase and peroxiredoxin, are expressed at high levels in K562 cells. We have further demonstrated that it is catalase, but not peroxiredoxin that plays a pivotal role in determining the cellular sensitivity to As_2_O_3_ and the up-regulated expression of catalase and peroxiredoxin was BCR/ABL independent. This study reveals that the functional role of antioxidant enzymes is cellular context dependent and catalase targeting compounds may be used in combination with As_2_O_3_ in CML treatment.

## Materials and Methods

### Cell culture

The ATRA-sensitive APL cell line, NB4, was obtained from Dr. Michel Lanotte (Hospital Saint Louis, Paris, France) [19]. The chronic myelogenous leukemia derived K562 cells were obtained from ATCC. 32D^MIGR1^ (a murine IL-3-dependent myeloid cell line transformed with empty retroviral Mig vector) and 32D^BCR/ABL^ (32D cells transformed to overexpress p210BCR/ABL) cells were established as previously described [20]. Cells were grown in RPMI-1640 (Bio-Whittaker Europe, Verviers, Belgium), supplemented with 10% fetal calf serum (FCS, EuroClone, Life Science Division, Milan, Italy) at 37°C in a humidified atmosphere of 5% CO_2_. The parental cell line 32D, 32D^MIGR1^ culture medium was supplemented with 1 U/mL recombinant mouse interleukin 3 (IL-3) (Strathmann Biotec, Hamburg, Germany). 32D^BCR/ABL^ cells are growth factor-independent. ATRA and arsenic trioxide (As_2_O_3_) were purchased from Sigma-Aldrich (St Louis, MO). A 100 mmol/L stock solution of As_2_O_3_ was obtained by dissolving As_2_O_3_ in 1 mol/L NaOH and dilution in H_2_O.

### Determination of cellular proliferation and apoptosis

The total number of cells and cell viability were determined by the trypan blue exclusion test (Sigma). Apoptotic cells in the populations were measured with a FACScan flow cytometer (Becton-Dickinson, San Jose, CA, USA) with the Annexin V FLUOS Apoptosis detection kit (Roche Molecular Biochemicals, Mannheim, Germany) according to manufacturer’s instruction.

### Detection of intracellular ROS

The oxidation-sensitive fluorescent probe dye, 2′,7′-dichlorodihydrofluorescein diacetate (DCF-DA, Invitrogen Molecular Probes, Eugene, OR) was used to measure the intracellular ROS concentration. DCF-DA is deacetylated intracellularly by nonspecific esterases and is further oxidized by cellular peroxides to the fluorescent compound 2′,7′-dichlorofluorescein. Briefly, cells treated with As_2_O_3_ or untreated cells were washed with phosphate buffered saline (PBS) and incubated with 20 µM DCF-DA at 37°C for 30 min according to the manufacturer’s instructions. The fluorescence signals were detected by a FACScan flow cytometer (Becton-Dickinson). For each sample, 5,000 or 10,000 events were collected. Hydrogen peroxide levels were expressed in terms of mean fluorescence intensity.

### RNA Interference and Transfection

Pairs of complementary oligonucleotides (Table S1) against PRDX 1, PRDX 2, PRDX 6, catalase, and non-target control shRNA (NC) were synthesized by Sangon Biotech (Shanghai, China), annealed, and ligated into the PSIREN-RetroQ Vector (Clontech Laboratories, Inc., CA, USA), respectively. The shRNA-carrying retroviruses, which were produced in 293T cells, were used to infect K562 cells.

### Western Blot Analysis

Cells were washed with PBS and lysed with lysis buffer (62.5 mM Tris-HCl, pH 6.8, 100 mM DTT, 2% SDS, 10% glycerol). Cell lysates were centrifuged at 20, 000 *g* for 10 min at 4°C, and proteins in the supernatants were quantified. Protein extracts were equally loaded onto an 8% to 14% SDS–polyacrylamide gel, electrophoresed, and transferred to nitrocellulose membrane (Amersham Bioscience, Buckinghamshire, UK). The blots were stained with 0.2% Ponceau S red to ensure equal protein loading. After blocking with 5% nonfat milk in PBS, the membranes were probed with antibodies against poly (ADP [adenosine diphosphate]–ribose) polymerase (PARP; 1∶500; Santa Cruz Biotech, Santa Cruz, CA), PRDX 1, PRDX 2, PRDX 3, PRDX 4, PRDX 5, PRDX 6, and Sirt1 (1∶1000; Santa Cruz Biotech), and followed by horseradish peroxidase (HRP)–linked secondary antibodies (Cell Signaling). The signals were detected by Chemiluminescence phototype-HRP kit (Cell Signaling) according to manufacturer’s instructions. As necessary, blots were stripped and re-probed with anti-actin or anti-tubulin antibodies (Oncogene, Fremont, CA) as an internal control. The signal intensity of the bands was quantified using Quantity One Version 4.4.0 (Bio-Rad). All experiments were repeated three times.

### Statistical Analysis

Student’s t-test was used to evaluate the difference between the two different treatments. A p value of less than 0.05 was considered statistically significant.

## Results

### K562 cells are more resistant to As_2_O_3_ treatment and have lower ROS levels than NB4 cells

**Figure 1:**
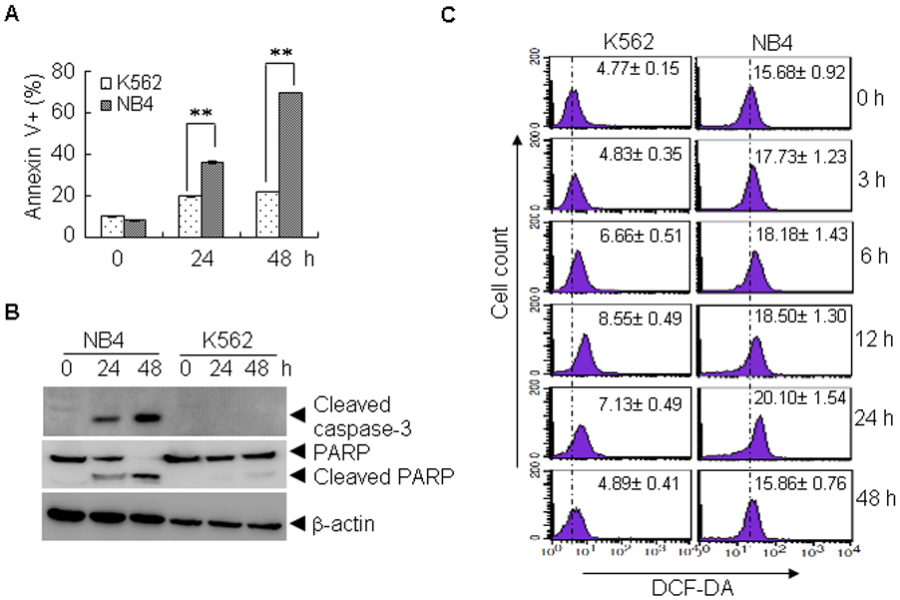
K562 cells have lower ROS levels and are more resistant to As_2_O_3_ treatment than NB4 cells. (A, B) K562 and NB4 cells were treated with As_2_O_3_ (2 µM) for 24 or 48 h, and the cell viability was determined by annexin V/PI staining (A), the indicated proteins were determined by western blot (B). All values represent means with bar as S.D. of three independent experiments, each of which with triplicate samples. (**p<0.01, compared to controls). (C) Untreated and As_2_O_3_-treated K562 and NB4 cells were stained with DCF-DA, the level of ROS was determined by FACS. All values represent means ± S.D. of three independent experiments.

The human K562 cell line was established from a chronic myelogenous leukemia patient that expresses the BCR/ABL fusion gene. The NB4 cell line was established from an acute promyelocytic leukemia patient that expresses the PML-RARα fusion protein. To compare the sensitivity of K562 and NB4 cells to As_2_O_3_, both cell lines were treated with 2 µM As_2_O_3_, a clinically relevant concentration, for 24 and 48 h. As shown in Figure 1A, an increased percentage of annexin V positive cells was observed in NB4 cells compared to K562 cells (Figure 1A). Accordingly, increased activation of caspase-3 and cleavage of PARP1 was observed in NB4 cells (Figure 1B). Because the effectiveness of As_2_O_3_ has been attributed to the induction of ROS [21], we examined the ROS levels in NB4 and K562 cells. Indeed, As_2_O_3_ treatment increases the ROS levels in both cell lines in a dynamic manner. As shown in Figure 1C, As_2_O_3_ treatment led to elevation of ROS in K562 cells as early as 3 h, peaked at 12 h, and then gradually decreased to the basal level at 48 h. Similar changes were observed in NB4 cells. Interestingly, the basal level of ROS in K562 cells is much lower than that in NB4 cells (4.77±0.15 vs. 15.68±0.92, p<0.05) (Figure 1C). These data suggest that the relative resistant to As_2_O_3_ treatment in K562 cells may be due to its ability to maintain a reduction status.

### Differential expression of peroxiredoxin and catalase in K562 and NB4 cells

**Figure 2:**
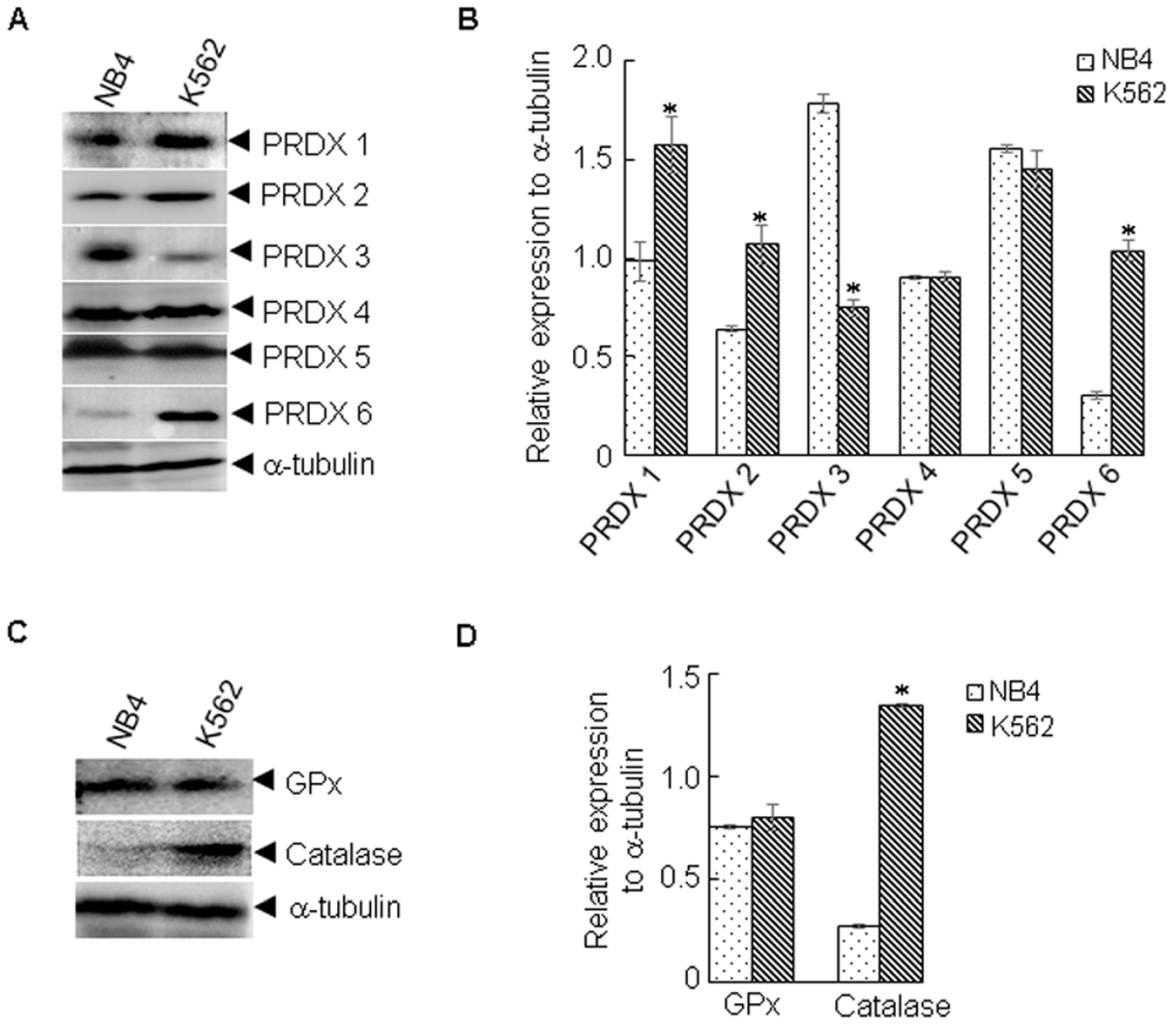
Differential expression of several antioxidant enzymes. K562 and NB4 cells were lysed and subjected to western blot. The indicated proteins were determined by western blot (A, C). The signal intensity of the indicated proteins against α-tubulin was quantified by Quantity One (B, D). All values represent means with bar as S.D. of three independent experiments. *p<0.05, compared to NB4 cells.

The homeostasis of redox status in cells is primarily controlled by antioxidant enzymes, such as catalase, glutathione peroxidase 1 (GPx), and PRDXs [22]. To examine whether the lower level of ROS observed in K562 cells was associated with these proteins, we compared their protein levels in K562 and NB4 cells. Peroxiredoxins (PRDXs) are highly conserved proteins, which include 6 family members, and function primarily to scavenge hydrogen peroxide (H_2_O_2_) [22]. Interestingly, four of six PRDX family members, PRDX 1/2/3/6, showed differential expression in K562 and NB4 cells. As shown in Figure 2A–B, PRDX 1/2/6 were up-regulated and PRDX 3 was down-regulated. Moreover, catalase was also up-regulated in K562 cells (Figure 2C, D). No significant difference in GPx was observed (Figure 2C, D). These data indicate that PRDXs and catalase might play a role in determining the ROS levels and cellular sensitivity to As_2_O_3_ in K562 cells.

### PRDXs knockdown cannot significantly alter the cellular sensitivity to As_2_O_3_

**Figure 3:**
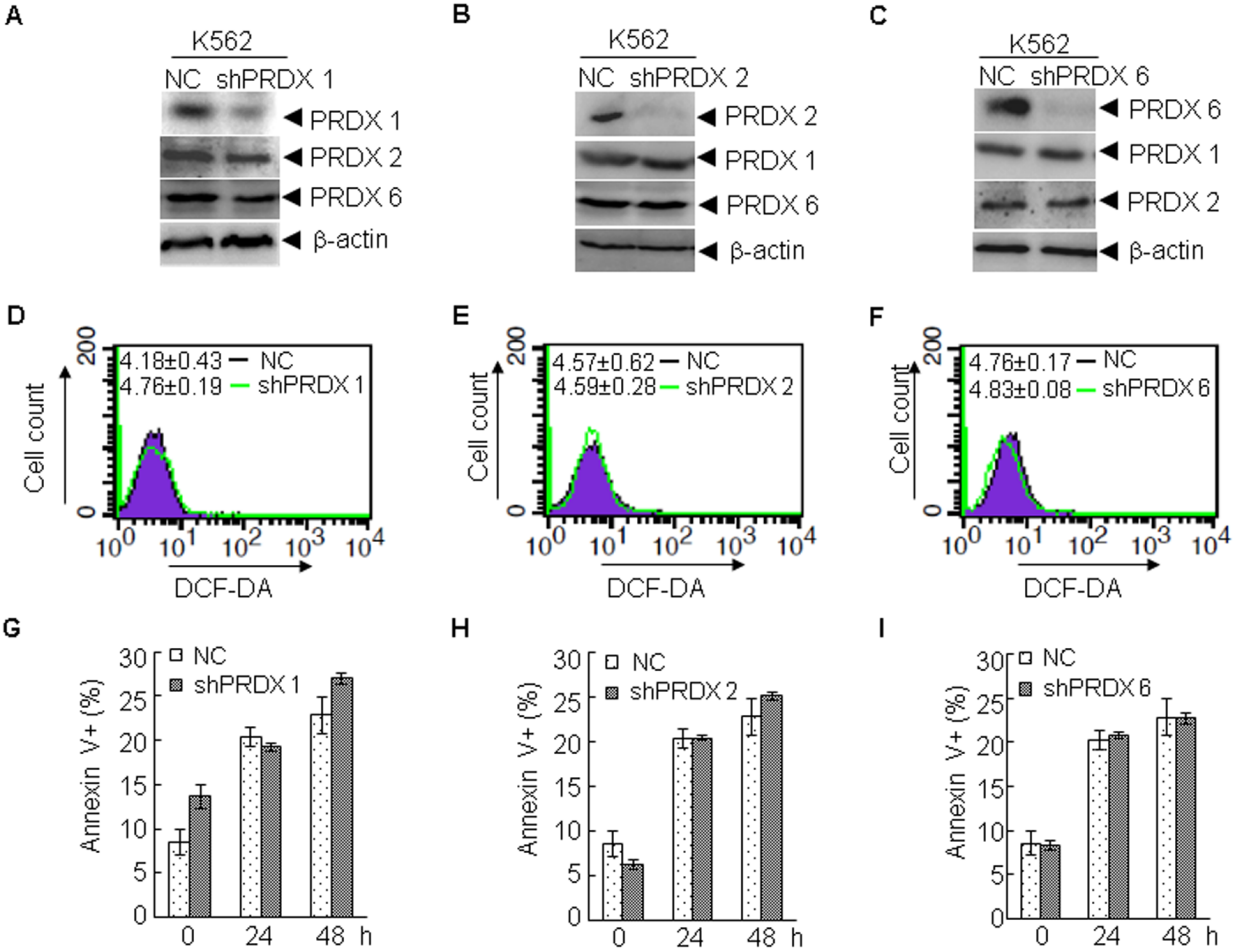
Knockdown of PRDX 1, 2, and 6 does not alter As_2_O_3_-induced apoptosis in K562 cells. (A–F) ShRNA against PRDX 1 (A), PRDX 2 (B), PRDX 6 (C) or non-specific shRNA were stably transfected into K562 cells, and the indicated proteins were examined by western blot (A–C), and the level of ROS (mean ± S.D.) was determined by FACS (D–F). Thereafter, the cells were treated with As_2_O_3_ for 24 or 48 h. Cell viability was determined by annexin V/PI staining (G–I). All values represent means with bar as S.D. of three independent experiments.

To test whether PRDXs contribute to the cellular resistance to As_2_O_3_ treatment, shRNAs specific to PRDX 1 (K562^shPRDX 1^), PRDX 2 (K562^shPRDX 2^), PRDX 6 (K562^shPRDX 6^), and the control shRNA (K562^NC^) were stably transfected into K562 cells. As shown in Figure 3A, protein levels of PRDX 1, but not PRDX 2 or PRDX 6, were specifically down-regulated by shRNA against PRDX 1. Similarly, PRDX 2 (Figure 3B) and PRDX 6 (Figure 3C) were also specifically silenced. We next determined the effect of PRDX 1/2/6 knockdown on ROS levels in K562 cells. However, PRDX 1/2/6 knockdown did not significantly increase the ROS levels in K562 cells (Figure 3D–F). Next, these cells were treated with As_2_O_3_ for 24 and 48 h. Compared with K562^NC^ cells, no significant changes in the percentages of annexin V positive cells were observed in K562^shPRDX 1^, K562^shPRDX 2^, or K562^shPRDX 6^ cells (Figures 3G–I). These results suggest that PRDX 1/2/6 may not play an important role in determining the sensitivity of K562 cells to As_2_O_3_.

### Knockdown of catalase increases the cellular sensitivity to As_2_O_3_

**Figure 4:**
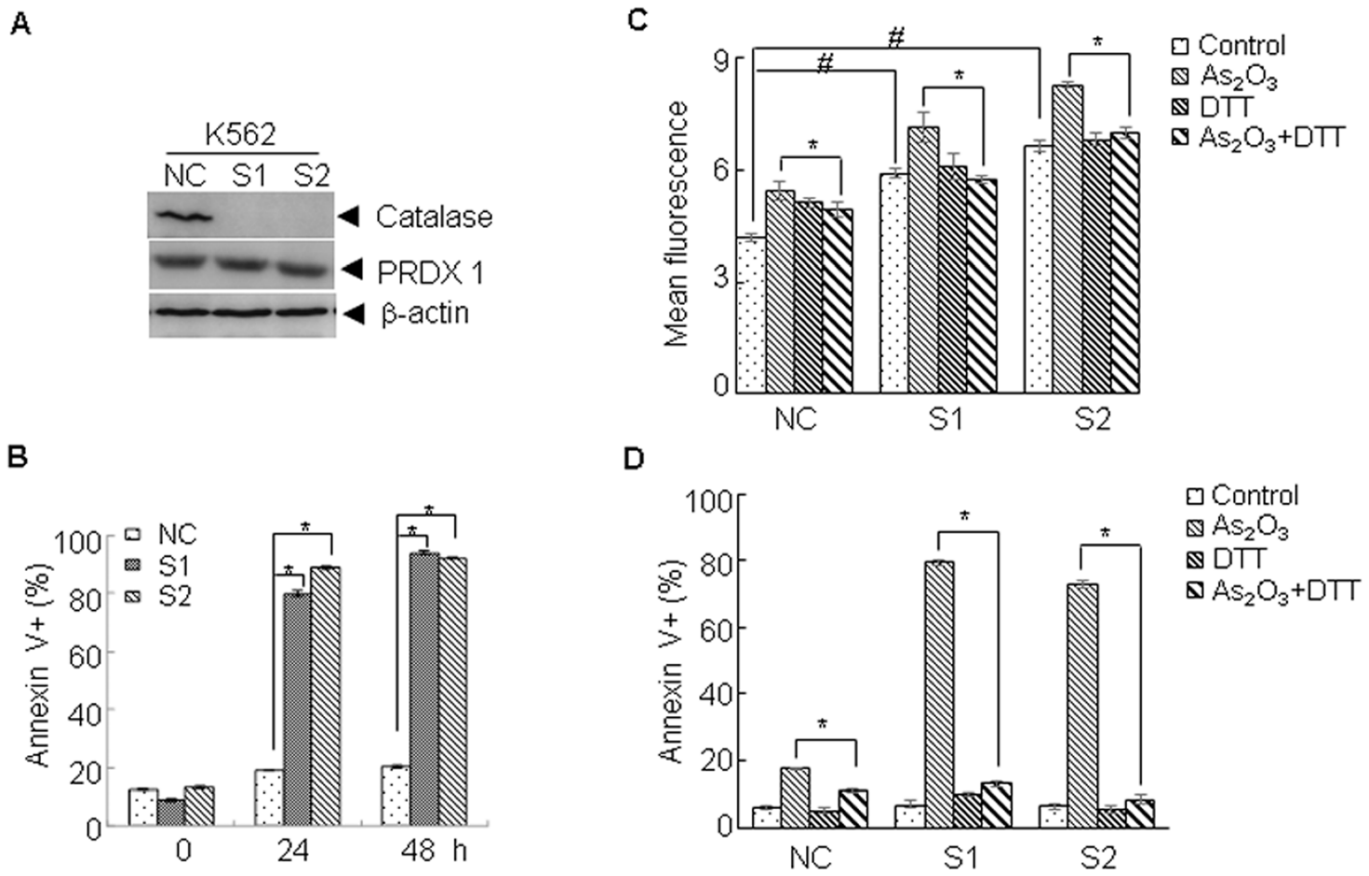
Knockdown of catalase enhances As_2_O_3_-induced apoptosis. (A, B) ShRNA against catalase or non-specific shRNA was stably transfected into K562 cells (NC for K562^NC^, S1 for K562^S1^, S2 for K562^S2^), the indicated proteins were determined by western blot (A). Thereafter, the cells were treated with As_2_O_3_ for 24 and 48 h, and the cell viability was determined by annexin V/PI staining (B). (C, D) The indicated cells were treated with As_2_O_3_ in the presence or absence of DTT (0.2 mM), then the level of ROS (12 h) was determined by FACS (C) and the cell viability (24 h) was determined by annexin V/PI staining (D). All values represent means with bar as S.D. of three independent experiments. (*p<0.05, #p<0.05).

We next investigated the possible role of catalase in the cellular sensitivity to As_2_O_3_. To this end, non-specific shRNA and specific shRNA against catalase were transfected into K562 cells. As shown in Figure 4A, catalase was specifically knocked down in K562 cells (K562^S1^ and K562^S2^). Compared with K562^NC^, knockdown of catalase did not directly induce apoptosis; however, sensitized K562 cells to As_2_O_3_ induced apoptosis, as evidenced by the significant increase in the percentage of annexin V positive cells (Figure 4B, p<0.05). To determine the role of ROS in the catalase knockdown induced sensitization of K562 cells to As_2_O_3_, the ROS levels in K562^NC^, K562^S1^, and K562^S2^ cells treated with or without As_2_O_3_ were determined by DCF-DA staining. As shown in Figure 4C, compared with K562^NC^, silencing of catalase could increase the ROS levels in K562^S1^, and K562^S2^ cells (p<0.05), which was further enhanced by As_2_O_3_ treatment. In the presence of DTT, a ROS scavenger, As_2_O_3_-induced ROS increasing was abrogated. Accordingly, DTT could significantly inhibit As_2_O_3_-induced cell death in K562^NC^, K562^S1^, and K562^S2^ cells (Figure 4D, p<0.05). These data suggest that catalase plays a critical role in determining the sensitivity to As_2_O_3_ in K562 cells through a ROS dependent manner.

### Effect of BCR/ABL on the expression of differential proteins

**Figure 5:**
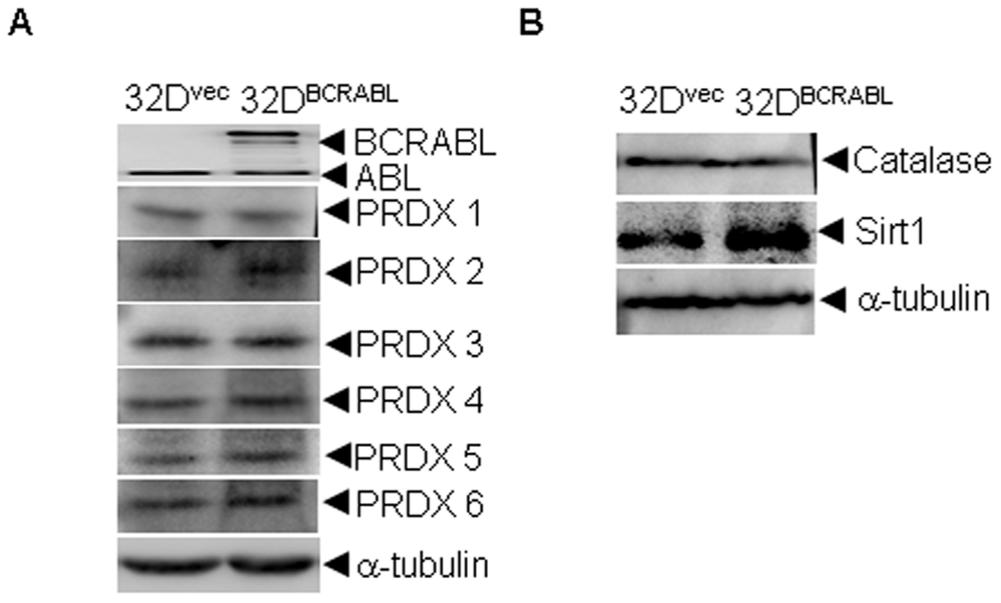
Overexpression of BCR/ABL on the expression of some antioxidant proteins. (A, B) 32D cells were stably transfected with BCR/ABL or the empty vector and the indicated proteins were examined by western blot. All experiments were repeated three times.

It has been established that ectopic expression of BCR/ABL in 32D cells, a murine IL-3-dependent myeloid cell line, results in resistance to apoptosis [23]. To determine whether the differential expression of antioxidant proteins observed between K562 and NB4 cells are due to the expression of BCR/ABL, we transfected a BCR/ABL plasmid into 32D cells and examined its effect on the expression of PRDX 1/2/3/6, catalase and Sirt1, a reported BCR/ABL up-regulated protein, by western blot analysis. As shown in Figure 5, except the up-regulation of Sirt1, protein levels of PRDX 1/2/3/6 and catalase were not altered by overexpression of BCR/ABL. These data suggest that the relatively higher expression of PRDXs and catalase observed in K562 cells is not directly related to overexpression of BCR/ABL.

## Discussion

K562 cells have a lower level of ROS than NB4 cells and it has been demonstrated that ROS has been linked with the toxicity of As_2_O_3_. In order to identify the possible factors determining the differential sensitivity to As_2_O_3,_ we examined the expression of several antioxidant enzymes in both cell lines. We demonstrated that catalase, but not PRDX family members, play an important role in determining the cellular susceptibility to As_2_O_3_ in K562 cells. We propose that targeting catalase may present a promising strategy to improve the efficacy of As_2_O_3_ in the treatment of CML.

PRDXs are a family of 6 ubiquitous peroxidases that reduce peroxides and their major functions, including protection against oxidative stress, induction of cell signaling and proliferation [24]–[25]. Aberrant high expression of PRDXs has been found in various kinds of cancers and contributes to chemotherapy or radiotherapy resistance [25]–[26]. However, their roles in leukemia are not well understood. PRDX proteins have variable expression levels in leukemia, suggesting disparity in functional significance depending on the cellular context. For example, PRDX 2 induction in Molt-4 cells was protective against apoptosis induced by etoposide treatment [27]; however, forced PRDX 2 expression could also result in decreased leukemogenesis in a transplantation AML mouse model [28]. Although it is known that oxidative stress induced by As_2_O_3_ play an important role in its cytotoxicity, the relationship between PRDXs and As_2_O_3_ sensitivity in leukemia cells has not been extensively studied [21]. In this work, higher expression of PRDX 1/2/6 and lower expression of PRDX 3 was observed in K562 cells compared to NB4 cells. As PRDX 1 can interact with BCR/ABL, and elevation of PRDX 1 has been observed in CML, we postulated that the higher expression of PRDX 1 in K562 cells may confer resistance to As_2_O_3_. Unexpectedly, knockdown of PRDX 1 did not sensitize the K562 cells to As_2_O_3_ treatment. Similarly, knockdown of PRDX 2/6 did not sensitize the K562 cells to As_2_O_3_ treatment, either. These results emphasize that the functional role of PRDXs is cellular context and treatment agent dependent. Consistent with this concept, we recently demonstrated knockdown of PRDX 1/2 can induce differentiation in NB4 cells [29]. Vivas-Mejia et al. found that down-regulation of PRDX3 resulted in increased sensitivity to As_2_O_3_ in NB4 cells [30]. Taken together, these data suggest that PRDX 1/2/6 may not play an important role in As_2_O_3_-induced cell death in K562 cells. Further studies are needed to elucidate a definite role of PRDXs in different kinds of leukemias.

Catalase can protect cells from the accumulation of hydrogen peroxide through decomposition of hydrogen peroxide into water and oxygen. However, overexpression of catalase does not necessary result in cell resistance to chemotherapy. For example, Sperana et al. demonstrated that catalase overexpressing cells are sensitized to bleomycin and paraquat exposure [31]. They proposed that catalase may be beneficial or harmful in different cell lines. Indeed, both overexpression and suppression of catalase have been observed in leukemia cells. Despite several reports showing that catalase also confers resistance to As_2_O_3_ in AML cells [30], [32]–[33], it is not known whether catalase plays an important role in As_2_O_3_-induced cell death in CML cells. In contrast to PRDXs, depletion of catalase markedly enhances As_2_O_3_-induced apoptosis, indicating that catalase play a critical role in As_2_O_3_-induced apoptosis in K562 cells. Combined our results with a previous report, which demonstrated high catalase activity in circulating cells from untreated CML [34], we propose that targeting catalase may improve the efficacy of As_2_O_3_ in CML cells.

In addition to directly phosphorylating its downstream substrates, BCR/ABL can transcriptionally activate several survival factors, such as Sirt1 [35]. Thus, the differential expressed proteins observed might be related to the expression of BCR/ABL. However, except for Sirt1, the protein levels of the PRDXs and catalase were not altered by overexpression of BCR/ABL. As K562 cells were established from a CML patient in blast crisis, the high expression of redox enzymes might due to other BCR/ABL independent secondary alterations. Thus, targeting these non-BCR/ABL regulated proteins in combination with BCR/ABL targeting agents, such as As_2_O_3_ or imatinib, may represent a novel strategy to treat CML.

In conclusion, we provide evidence that the peroxiredoxin protein may not play an essential role in determining the cellular susceptibility to As_2_O_3_, while targeting catalase may greatly enhance the efficacy of As_2_O_3_ in CML therapy.

## Supporting Information

Table S1 Sequences for shRNA plasmids construction.
(DOC)
