# Supplementary material for: Targeting Catalase but Not Peroxiredoxins Enhances Arsenic Trioxide-Induced Apoptosis in K562 Cells
Source: PLoS One. 2014 Aug 12;9(8):e104985. doi: 10.1371/journal.pone.0104985 (PMC4130628; doi:10.1371/journal.pone.0104985)
Supplement: Table S1 — Sequences for shRNA plasmids construction. (DOC) [file pone.0104985.s001.doc]

**Targeting Catalase but not Peroxiredoxins Enhances Arsenic Trioxide-induced Apoptosis in K562 Cells**

Li-Li Song^#^, Yao-Yao Tu^#^, Li Xia^#^, Wei-Wei Wang, Wei Wei, Chun-Min Ma, Dong-Hua Wen, Hu Lei, Han-Zhang Xu*****, Ying-Li Wu*****

**Supplementary Table S1 Sequences for shRNA plasmids construction**

| Knockdown constructs | Target sequences (5'-3') |
| --- | --- |
| NC (negative control) | TCCCGTGAATTGGAATCCT |
| shRNA-PRDX 1 (shPRDX1) | CTGCCAAGTGATTGGTGCT |
| shRNA-PRDX 2 (shPRDX2) | TTAGGCTGGCTAACGGATA |
| shRNA-PRDX 6 (shPRDX6) | AAGGTTTTTAGGTTGCTAT |
| shCatalase-1 (S1) | AGATGATCTACTCAGAAAT |
| shCatalase-2 (S2) | CCAGTAGGAGACAAACTTA |
